# Phosphatidylserine positive microparticles improve hemostasis in *in-vitro* hemophilia A plasma models

**DOI:** 10.1038/s41598-020-64686-x

**Published:** 2020-05-12

**Authors:** Yanan Zong, Iva Pruner, Aleksandra Antovic, Apostolos Taxiarchis, Zara Pons Vila, Nida Soutari, Fariborz Mobarrez, Roza Chaireti, Jerker Widengren, Joachim Piguet, Jovan P. Antovic

**Affiliations:** 1 Department of Molecular Medicine & Surgery, Karolinska Institutet, Stockholm, Sweden; 2 Department of Medicine, Division of Rheumatology, Karolinska Institutet, Stockholm, Sweden; 3 Academic Specialist Center, Center for Rheumatology, Stockholm Health Services, Stockholm, Sweden; 4 Coagulation, Clinical Chemistry, Karolinska University Hospital, Stockholm, Sweden; 5 Department of Medical Sciences, Uppsala University, Akademiska Hospital, Uppsala, Sweden; 6 Department of Hematology, Karolinska University Hospital, Stockholm, Sweden; 7 Experimental Biomolecular Physics, Department of Applied Physics, KTH Royal Institute of Technology, Stockholm, Sweden

**Keywords:** Haematological diseases, Translational research

## Abstract

Circulating microparticles (MPs) are procoagulant due to the surface containing phosphatidylserine (PS), which facilitates coagulation. We investigated if MPs improve hemostasis in HA plasma models. MPs isolated from pooled normal human plasma were added to severe, moderate and mild HA plasma models (0%, 2.5%, 20% FVIII). The MPs’ effect on hemostasis was evaluated by calibrated automated thrombogram (CAT) and overall hemostasis potential (OHP) assays, while fibrin structure was imaged by standard confocal, stimulated emission depletion (STED) microscopy and scanning electron microscopy (SEM). MPs partially restored thrombin generation and fibrin formation in all HA plasma models. The procoagulant effect of MPs requires PS exposure, to a less extent of contact pathway activation, but not tissue factor exposure or *in vitro* stimulation of MPs. MPs partially normalized the fibrin structure, and using super-resolution STED, MPs attached to fibrin were clearly resolved. In summary, our results demonstrate that PS positive MPs could improve hemostasis in HA plasma models.

## Introduction

Hemophilia A (HA) is an inherited bleeding disorder, which is caused by a deficiency in coagulation factor VIII (FVIII)^1^ and characterized by insufficient thrombin generation and fibrin formation^2^. Patients with HA are characterized as having: severe (<1% of normal FVIII activity), moderate (1–5%) or mild (5–40%) HA. Prophylactic FVIII replacement therapy is recommended for severe and some moderate HA patients, in order to transform the bleeding phenotype from severe to non-severe^3^. The major concerns of current therapy to HA are the development of FVIII inhibitors, and the need of frequent injections due to the short half-life time of drugs^4^. Moreover, because of the high cost of therapy, HA patients in many developing countries do not have access to prophylaxis^5^. Finding novel potential adjunctive therapies for HA is therefore of interest.

Circulating microparticles (MPs) are small (0.1–1 µm) membrane vesicles originating from many different cells by membrane blebbing after activation, apoptosis, or high shear stress^6^. MPs, as their parental cells, may provide cell surface component and participate in the coagulation process^7^. In *in vitro* studies with plasma from healthy individuals, MPs enhance thrombin generation, fibrin clot structure and clot stability^8,9^. Elevated levels of total MPs, especially tissue factor (TF) positive MPs, have been associated with cardiovascular disease and cancer^10^.

Few studies have investigated the role of MPs in HA. Levels of MPs in plasma have been found to be higher in untreated HA patients compared with healthy individuals^11^. One previous clinical study of plasma from on-demand-treated severe HA patients showed that the level of MPs decreased after FVIII treatment, and was inversely correlated with thrombin generation and fibrin formation. These findings suggest that MPs may participate in the formation of hemostatic clots in severe HA patients^12^. In an *in vivo* FVIII-knockout HA mouse model, a threefold increase in total MP level induced by soluble P-selectin infusion normalized the tail vein bleeding time^13^.

This study was aimed at investigating the contribution of MPs isolated from pooled normal human plasma (PNP) in improving hemostasis in *in vitro* HA models. The effects of MPs on thrombin generation, fibrin formation and clot structure were evaluated using global hemostatic tests, and imaging methods. Stimulated emission depletion (STED) microscopy was used to gain insight into the incorporation of MPs in fibrin networks.

## Results

### **Characterization of MPs by flow cytometry is shown in Supplementary data**

#### The effect of MPs on thrombin generation in *in vitro* HA plasma models

**Figure 1:**
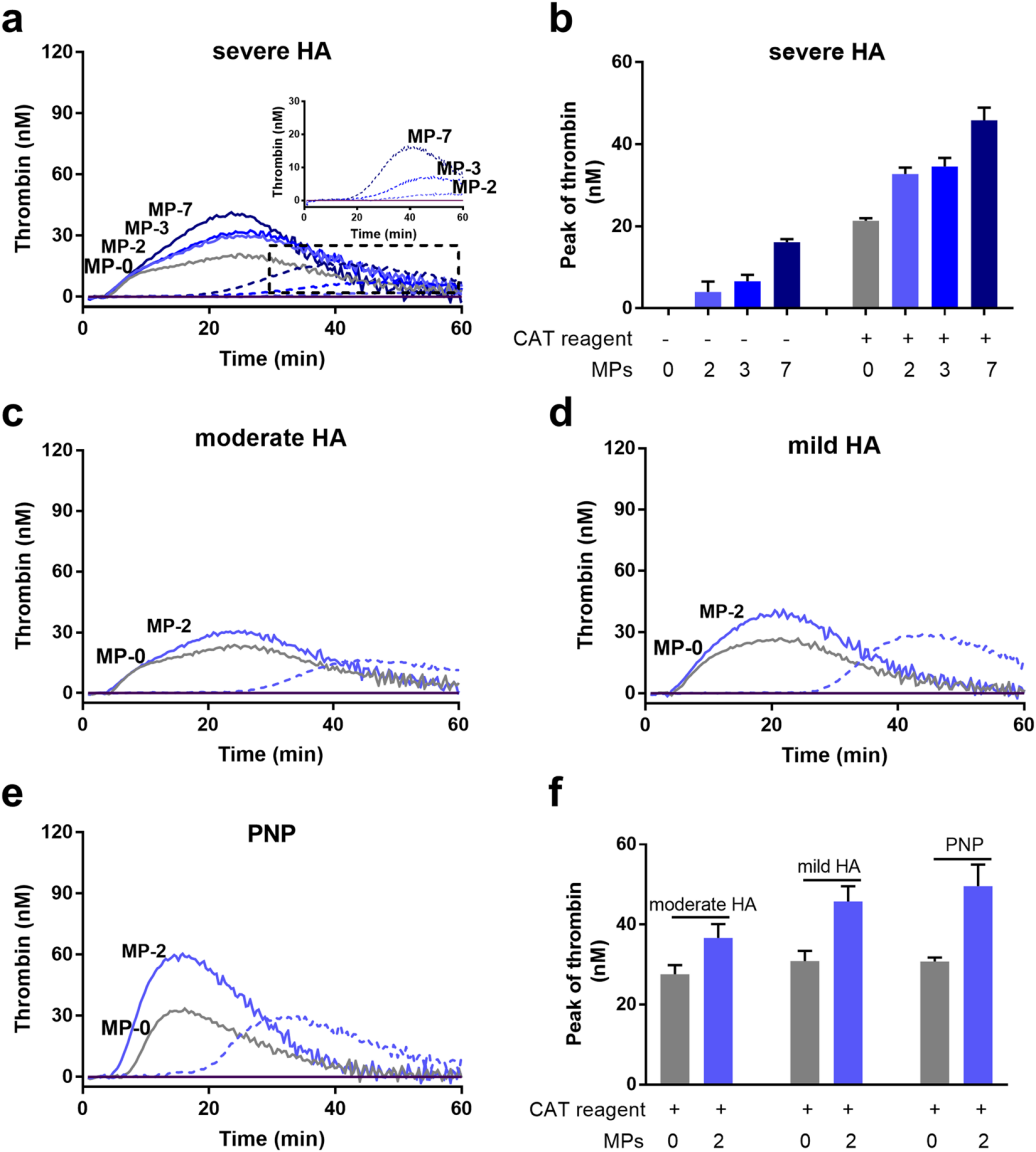
Isolated MPs improve thrombin generation in all HA plasma models and in PNP as detected by the CAT assay. (**a**) Thrombin generation in the severe HA plasma model with different concentrations of MPs (MP-0, 2, 3 and 7: 0, 2, 3 and 7 × 10^4^ MPs/µL plasma), in the presence (solid lines) and absence (dashed lines) of PPP-Reagent LOW (CAT reagent). The inset shows thrombin generation curves (with an adjusted y-axis scale) in the absence of CAT reagent. (**b**) Peak thrombin value in the severe HA plasma model. (**c–e**) Thrombin generation in other plasma models with MPs (2 × 10^4^ MPs/µL plasma) in the presence (solid lines) and absence (dashed line) of CAT reagent: (**c**) moderate HA (2.5% FVIII); (**d**) mild HA (20% FVIII), and (**e**) PNP (100% FVIII). (**f**) Peak thrombin value in the moderate, mild HA plasma models and in PNP. In all plasma models, without MPs and without CAT reagent, the thrombin generation curves were flat at baseline level. Data shown are mean ± SEM values, n = 9 replicates.

In the severe HA model, MPs increased peak thrombin generation in a dose-dependent manner both in the presence (solid lines in Fig. 1a, and b) and absence (dash lines and inset in Fig. 1a, and b) of CAT reagent. The lag-time was also shortened by MPs dose-dependently in the absence of CAT reagent (dash lines and inset in Fig. 1a). The PBS control without MPs or CAT reagent showed no thrombin generation. Addition of MPs at a selected concentration (2 × 10^4^ MPs/µL) increased peak thrombin generation in the moderate (2.5% FVIII) and mild (20% FVIII) HA models and in PNP (Fig. 1c–f).

#### The effect of MPs on fibrin formation and clot stability in *in vitro* HA plasma models

**Figure 2:**
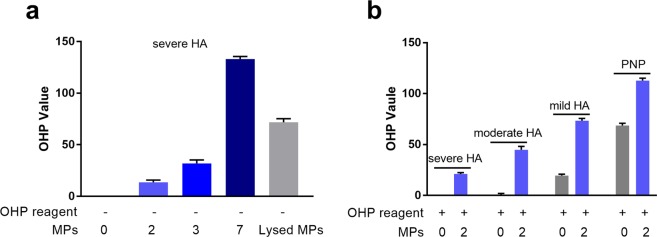
The effect of MPs on fibrin clot formation and clot stability in different HA plasma models. (**a**) In the severe HA plasma model, OHP values after addition of different concentrations of MPs (2, 3 and 7 × 10^4^ MPs/µL plasma) in the absence of OHP reagent are shown. Lysed MPs: MPs (7 × 10^4^ MPs/µL plasma) were treated with TritonX-100 (0.25% for 15 min at RT); (**b**) OHP values in different HA plasma models and in PNP, without and with MPs (2 × 10^4^ MPs/µL plasma) in the presence of OHP reagent. OHP value: area under the curve (absorbance vs. 2 h) of clot formation and fibrinolysis (with t-PA). Data are means ± SEM values, n = 9 replicates.

In the severe HA plasma model, addition of MPs increased the OHP values in the absence of OHP reagent (Fig. 2a). The OHP value achieved with the highest concentration of MPs (7 × 10^4^ MPs/µL plasma) decreased dramatically after lysing the MPs with 0.25% TritonX-100 (Fig. 2a). Without addition of MPs, the OHP values were negligible, and no fibrin clot was formed within 2 h. In the presence of OHP reagent, addition of MPs at a selected concentration (2 × 10^4^ MPs/µL) increased OHP values mostly in the severe and moderate HA model, but the OHP values were still lower than in the control plasma. In the mild HA model, addition of MPs increase the OHP value to a less extent, however, those values were comparable with the control plasma (Fig. 2b).

#### Contribution of PS, TF, contact activation and in vitro stimulation to the MP-associated procoagulant activity

**Figure 3:**
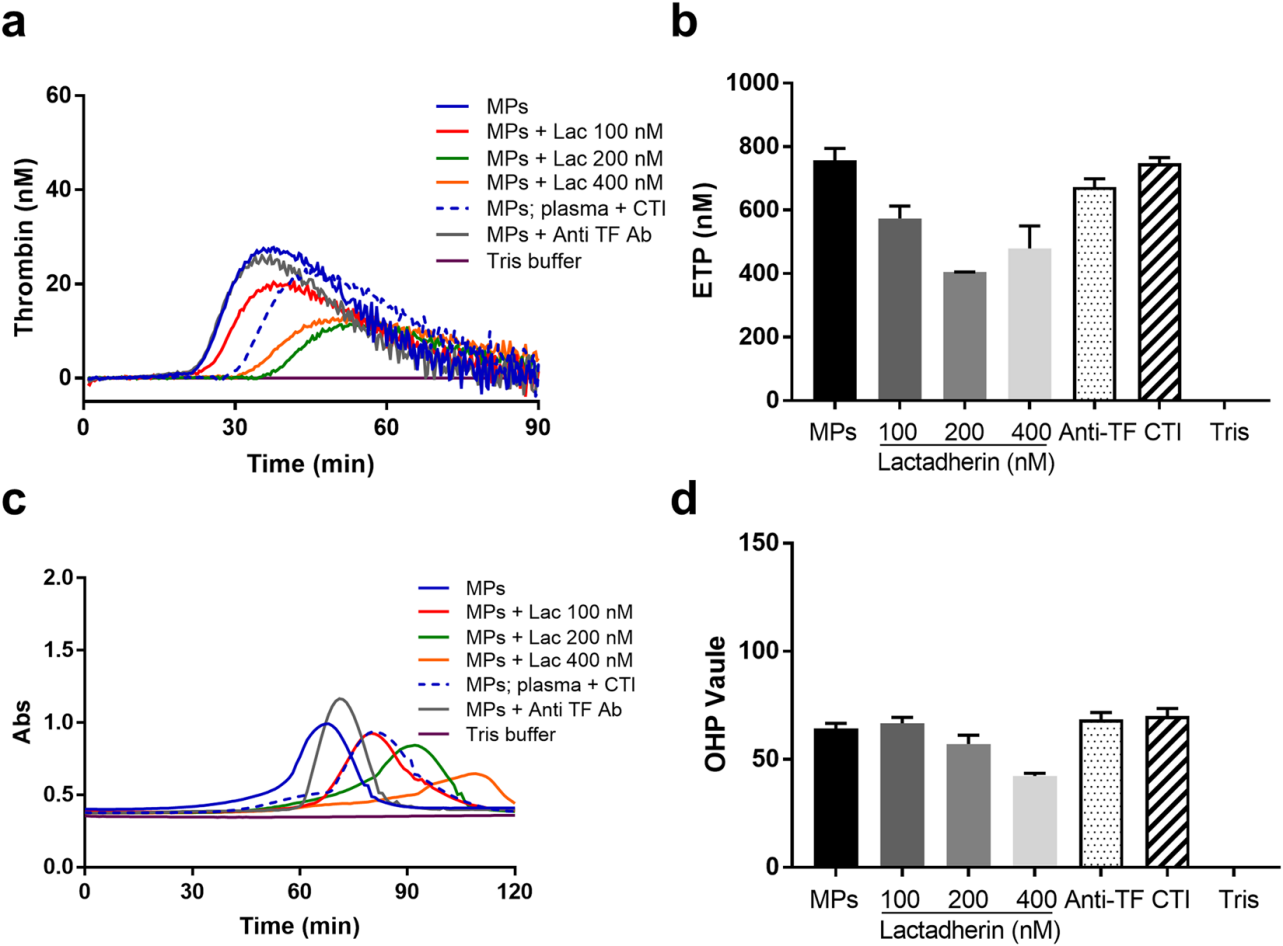
The procoagulant effect of MPs after blocking PS, TF or contact pathway activation in the mild HA plasma model and in the absence of CAT and OHP reagent. (**a**) Thrombin generation curves, (**b**) endogenous thrombin potential (ETP), (**c**) absorbance curves of fibrin clot formation and fibrinolysis and (**d**) OHP values. MPs were incubated with lactadherin (MPs + lac 100 nM, 200 nM and 400 nM) to block PS, anti-TF antibodies (MPs + Anti-TF) to block TF; and plasma was incubated with CTI before addition of MPs (MPs; plasma + CTI) to block contact activation. Tris buffer was used as negative control. Data are means ± SEM, n = 9 replicates.

The experiments were performed using the mild HA model, where the procoagulant activity of MPs was more evident. Pre-incubation of MPs with lactadherin inhibited thrombin generation in a dose-dependent manner: ETP, decreased by ~27.1%, 46.6% and 36.7% respectively, while the lag-time was prolonged up to 1.4-fold using the highest concentration of lactadherin (400 nM) (Fig. 3a,b). In contrast, pre-incubation of MPs with anti-TF antibodies did not influence the ETP (Fig. 3a,b). Blocking contact activation with CTI prolonged the lag-time slightly, but did not reduce the ETP value substantially (Fig. 3a,b**)**. Consistent with the CAT assay results, in the OHP assay pretreatment of MPs with lactadherin inhibited fibrin formation in a dose-dependent manner and the OHP value decreased by 34.2% with the highest concentration of lactadherin (Fig. 3c,d). Preincubation of MPs with anti-TF antibodies did not affect OHP results (Fig. 3c,d). Addition of CTI to block contact activation did not inhibit fibrin formation (Fig. 3c,d). The same concentration of PMPs (2 × 10^4^ MPs/µL plasma) with and without TRAP6 treatment induced similar levels of thrombin generation and fibrin formation (Supplementary data).

#### MPs’ effect on fibrin clot structure detected by confocal microscopy and SEM

**Figure 4:**
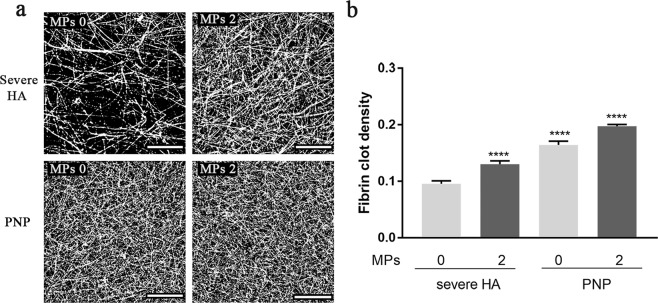
Standard confocal microscopy showed that MPs increased fibrin clot density in the severe HA plasma model. (**a**) Fibrin clot structure in the severe HA plasma model and PNP control, with (MPs 2) and without (MPs 0) addition of MPs (2 × 10^4^ MPs/µL plasma). Images shown are representative slices from optical sections (scale bar = 25 µm); (**b**) Fibrin clot density analyzed by using Fiji software. The clot samples were all prepared in the presence of OHP reagent. Data are means ± SEM, n = 3 fibrin clots (8 optical stacks/clot). ****p < 0.0001 compared to severe HA model without addition of MPs.

Using confocal microscopy, we found that MPs (2 × 10^4^ MPs/µL plasma) increased fibrin clot density in the severe HA model, suggesting that they improved the typical coarse and porous fibrin network (Fig. 4a,b).

**Figure 5:**
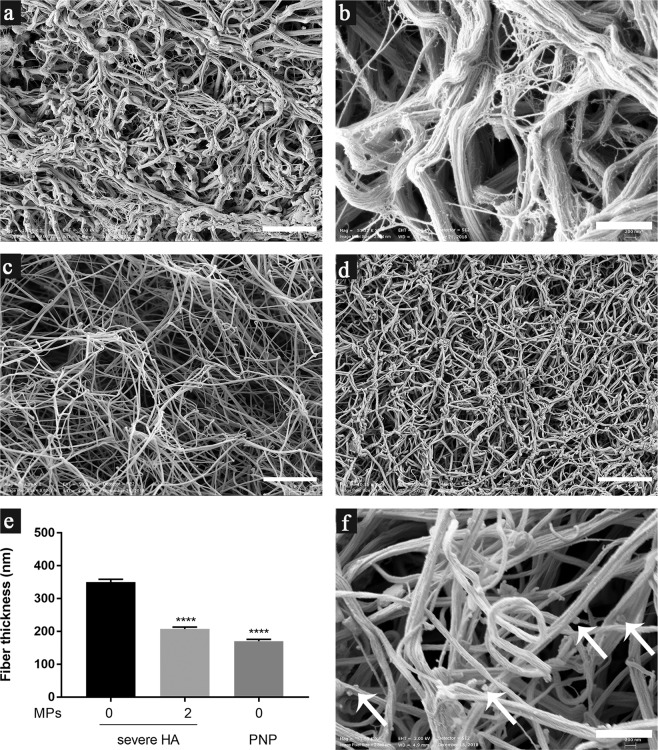
MPs improved fibrin clot structure in the severe HA plasma model as detected by SEM. (**a,b**) Fibrin clot structure in the severe HA plasma model without addition of MPs; (**c**) Fibrin clot structure in the severe HA model with MPs (2 × 10^4^ MPs/µL plasma) in the absence of OHP reagent; (**d**) Fibrin clot structure in the control PNP; (**e**) Fiber thickness (mean ± standard error) in fibrin clots in the severe HA model and PNP, n = 3 fibrin clots (100 individual fibers/clot), ****p < 0.0001 compared to severe HA model without addition of MPs; (**f**) Fibrin clot in the severe HA model with addition of PMPs (2 × 10^4^ PMPs/µL plasma) isolated from platelet concentrate without TRAP6 treatment. The arrows indicate potential MPs attached to fibers. Bar = 5 µm in (**a**,**c**, and **d**); and bar = 1 µm in (**b** and **f**).

The SEM images of a clot from the severe HA plasma model without addition of MPs showed coarse fibrin networks with thick fibers (Fig. 5a,b). Thin fibrin structures were also revealed. However, these were not individual fibers and were likely to be structures that had failed to become assembled into fibers (Fig. 5b). Fibrin clot structure was partially normalized by addition of MPs (2 × 10^4^ MPs/µL plasma): fibrin fibers became thinner and were intertwined randomly (Fig. 5c,e). In a clot from the severe HA model with addition of MPs, fiber thickness was similar to that in the control PNP, although pores among fibers were larger than those in the normal plasma model, suggesting that addition of MPs did not fully restore normal fibrin structure (Fig. 5c vs. d).

We did not see any “MP-like structures” in fibrin clots from the severe HA model **(**Fig. 5c). Interestingly, in a fibrin clot with addition of PMPs (obtained from platelet concentrate without TRAP6 treatment), discrete granular structures (100–200 nm) were found attached to the fibers, and were suspected to be MPs (Fig. 5f, arrows). Notably, the MP-like structures were found only in a small portion of the fibrin clot, which is one limitation of the SEM technique, i.e. limited sampling.

#### Super-resolution STED imaging of MPs attached to fibrin fibers

Using first diffraction-limited (standard) confocal microscopy, we found small particles with specific CD61 staining (MPs) incorporated in the 3-dimentional fibrin network (Supplementary data). However, we were not able to confirm whether those MPs were attached to fibrin fibers, as the diameters of the majority of the particles and the fibers were below the resolution limit of confocal microscopy (200–300 nm)^14^. To better resolve the location of MPs in the fibrin network, we used STED imaging. Fiber thickness measured in STED imaging ranged from 100 to 400 nm, which is consistent with the SEM measurements, while the diameter of the MPs ranged between 80 to 800 nm. The sizes of the MPs were not evenly distributed. Two sub-populations were observed, one with diameters smaller than 400 nm, and the other with diameters larger than 600 nm. This can be explained by aggregation of MPs at specific locations, or by the actual existence of subpopulations. Notably, small MPs that are close to each other can be distinguished in STED images, while they would be incorrectly identified as one large MP in standard confocal microscopy (Supplementary data).

**Figure 6:**
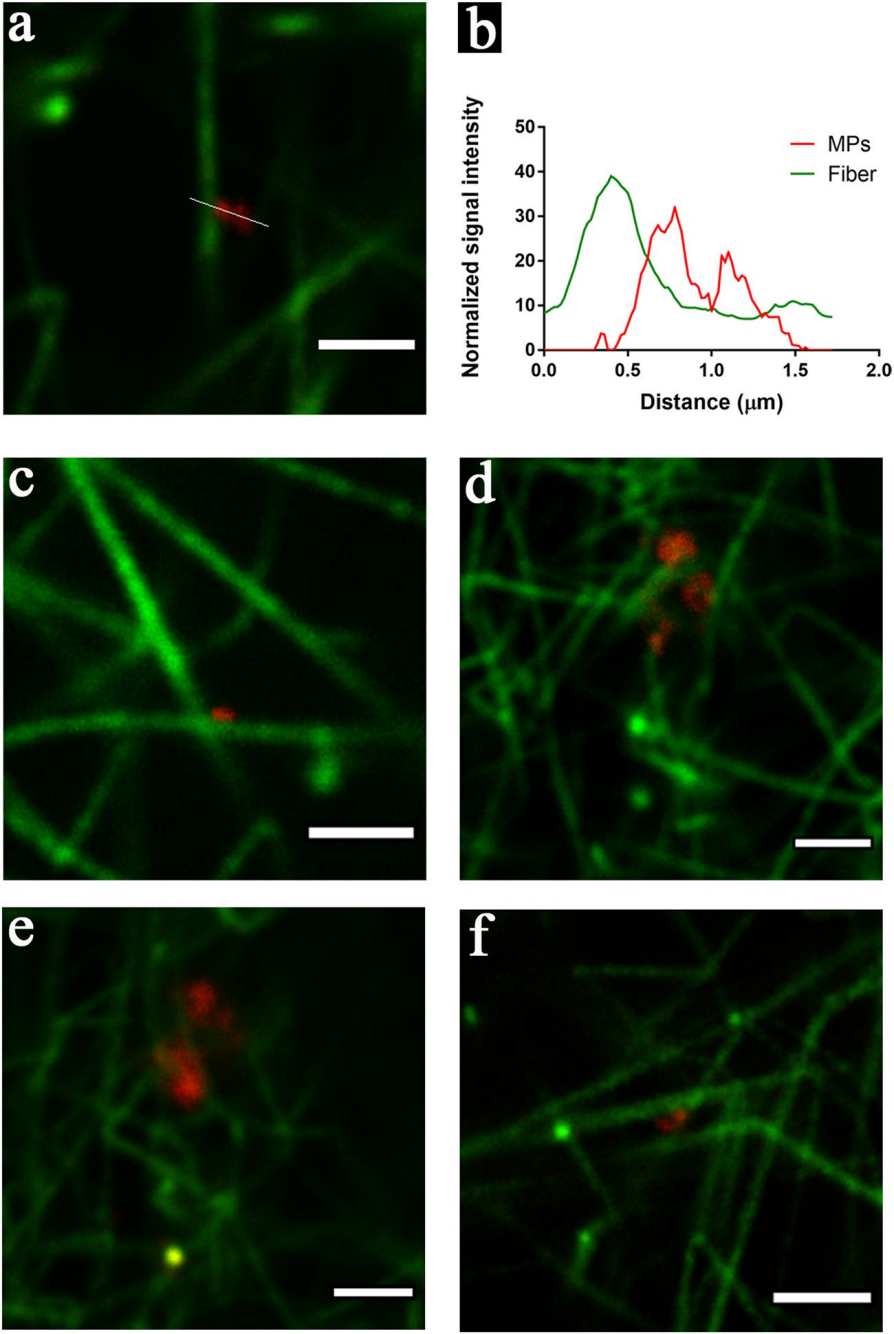
PMPs incorporated in the fibrin network detected by STED. (**a**) PMPs attached to a fibrin fiber; green: fiber (Alexa fluor 594), red: PMPs (Aberrior STAR 635); (**b**) The corresponding intensity profile along the line shown in (**a**), intensity normalized on the basis of the minimum intensity value in each channel; (**c,d**) Different typical patterns of the location of PMPs in the fibrin network: (**c**) PMPs located at the cross point of two fibers; (**d**) Fiber turned direction near the attached PMPs; (**e**) PMPs embedded in a mesh of fibrin fibers; (**f**) PMPs act as a bridge connecting two fibers. Images are all from the severe HA plasma model with addition of MPs. Representative images from two independent experiments are shown; duplicate samples/experiment. Bar = 2 µm.

No PMPs were visualized in the fibrin clot without addition of MPs (not shown). In the fibrin clots with addition of MPs, in both the severe HA model and in the control plasma, PMPs were found to be attached to fibrin fibers (Fig. 6a). There was no spatial overlap between the PMPs and the fibers in the STED images, which indicates that PMPs are bound to the fibers via interaction between their surfaces and the fibrins (Fig. 6b). Interestingly, PMPs tended to be located near the junctions of overlapping fibrin fibrils (Fig. 6c), implying that PMPs mediate fiber–fiber interaction. Some fibers turned direction at the point where a PMP was attached (Fig. 6d). Fibers formed a mesh around the PMPs, suggesting that fibrin fibers might have started assembling near MPs (Fig. 6e). In some cases, PMPs acted as a bridge connecting two different fibers (Fig. 6f).

## Discussion

In the present study, we investigated the role of MPs in improving hemostasis in *in vitro* HA plasma models using global hemostatic assays and imaging techniques. We found that MPs partially increased thrombin generation, and improved fibrin formation and clot stability in a dose-dependent manner in the severe HA plasma model. The procoagulant activity of the MPs depended on the presence of PS on their surface, to a less extent on contact pathway activation, but not on the presence of TF or *in vitro* stimulation of MPs. Addition of MPs partially normalized fibrin structure in the severe HA model, as found by confocal microscopy and SEM. Furthermore, we confirmed by STED imaging that MPs were incorporated in the fibrin network, and that they were typically located at fiber junctions and branching points.

In our severe HA plasma model, we observed that MPs shifted the impaired thrombin generation and fibrin formation towards a moderate or mild HA pattern and that this effect is dose-dependent. After incubating MPs with TritonX-100, the OHP value decreased dramatically, suggesting that the procoagulant effect is due to the presence of intact membrane vesicles. The thrombin generation parameters and OHP values in the HA plasma models were all lower than in the control plasma, suggesting that addition of MPs can only partially but not fully compensate for FVIII deficiency. Addition of MPs to PNP increased thrombin generation and fibrin formation, which is consistent with the results of other studies^15–17^.

We have provided evidence that the procoagulant effect of MPs was independent of TF, since almost no TF + MPs were found in the MPs isolated from PNP as characterized using flow cytometry and incubating the MPs with anti-TF antibodies did not inhibit thrombin generation or fibrin formation in our HA plasma model. In contrast, the presence of intact PS + membrane surfaces on MPs is essential, as blocking of PS on MPs with lactadherin inhibited thrombin generation and fibrin formation substantially. Blocking contact activation using CTI reduced but did not completely abolish thrombin generation and fibrin formation, which is consistent with previous studies^15^. We found that PMPs isolated from platelet concentrates, with and without *in vitro* activation, had similar procoagulant activity. Similar to our finding, PMPs derived from platelets treated with different agonists have revealed similar prothrombinase activity^18^. This suggests that the amount of PS + membrane is crucial to the procoagulant activity of MPs, regardless of the PMP derivation method.

We suggest that MPs possess similar procoagulant properties as activated platelets by expressing PS + membrane as described in cell-based coagulation model^19^. Confocal microscopy studies have shown that FVa and FXa colocalize on MPs^20,21^. In the severe HA model, the propagation phase of coagulation activation is impaired due to the deficiency of FVIII as a cofactor, while the addition of MPs can enhance clotting propagation. MPs provide the phospholipid surface for the binding of FIXa and FX, and subsequently increase their effective concentrations, thereby accelerating the conversion of FX to FXa^22^. We found that MPs improved thrombin generation and fibrin formation in the presence of CAT or OHP reagent, which contained small amounts of TF or thrombin. Since coagulation initiated by TF or thrombin resembles the *in vivo* conditions of patients at the site of injury, our results suggest that MPs could be considered as a supplement to treatment in patients with HA upon bleeding episodes.

Using confocal and scanning electron microscopy we found that in the severe HA model, MPs increased fibrin clot density, and the thick and coarse fibrin fibers were changed into thin and compact fibers by MPs. The results of these microscopy analyses is consistent with the OHP analysis, showing that addition of MPs improved fibrin formation in HA plasma model. Addition of MPs also improved fibrin formation in PNP, which is consistent with previous report. By comparing the fibrin formation in platelet-free and MPs-depleted plasma from the same healthy volunteer, Zubairova *et al*. found that MPs accelerated fibrin formation^9^. In contrast, Aleman *et al*. demonstrated that the fibrin density of normal plasma was not changed by adding PMPs^18^.

Circulating PMPs from healthy individuals, and from patients with thrombotic and other conditions, have been reported to be incorporated in fibrin clots in confocal microscopy and SEM studies^9,20,23^. In the present study, by using STED we showed for the first time the presence of PMPs in close proximity to fibrin fibers, and PMPs were attached to fibers particularly at branch points and junctions. These specific locations of PMPs in the fibrin network suggest that the fibrin network might be formed around the surface of PMPs, and that PMPs may participate in the distribution of fibrin fibers in the network. Platelets increase fibrin density by binding with high affinity to fibrin(ogen) in plasma via integrin on their cell surface^24^. One early study, based on immunolabeling SEM and X-ray microanalysis, revealed that both platelets and PMPs were incorporated in fibrin clots generated during whole-blood perfusion in a flow chamber^25^.

Our finding that MPs improves hemostasis in HA models and that MPs incorporate in fibrin clots may be of clinical importance. In future work, more research is needed to test and apply MPs as biomarkers for prediction of bleeding phenotype in HA, as well as a potential autologous adjunct therapy to improve replacement treatment.To conclude, our results demonstrated that MPs increased thrombin and fibrin generation, improved fibrin structure, and were incorporated in the fibrin network in HA plasma models. The procoagulant property of the MPs was found to be concentration-dependent, independent of tissue factor, and dependent on phosphatidylserine. MPs may participate in modifying the bleeding phenotypes in patients with HA. Additionally, autologous MPs may theoretically represent a potential adjunctive treatment in HA and are at least of interest to be further investigated in animal models.

## Materials and Methods

### Pooled normal human plasma and platelet concentrates

Pooled normal human plasma and platelet concentrates were obtained from the Department of Transfusion Medicine. This study was part of a larger study, which was approved by the regional ethics review board in Stockholm (Dnr 01–0003;2006/778–32, completed with 2013/1045–32, 2015/275–32 and 2018/1480–32). The study was conducted in accordance with the declaration of Helsinki. Informed consent were obtained from the participants. For pooled normal plasma, blood samples (30 mL/volunteer) were collected from healthy volunteers (n = 25; 18–65 years) with no history of bleeding or thrombotic diseases into 0.109 M citrated vacutainers (Becton Dickinson (BD), USA). Plasma was prepared within 4 hours of collection by centrifugation at 3000 × *g* for 10 min, at 15 °C, pooled and aliquoted (1 or 2 mL) into plastic tubes, and stored at −70 °C. Platelet concentrates were prepared from buffy coat (from blood) of 8 different healthy donors, pooled and kept in platelet additive solution (53–68%, PAS-E; and 32–47% plasma), with the concentration of 1300–1500 × 10^9^ platelets/L. The platelet concentrates were kept on agitator at 18–22 °C and used within 5 days after preparation.

### Preparation of HA plasma models

FVIII-deficient plasma (George King Bio-Medical, USA) was used as severe HA plasma model. The moderate and mild HA plasma models were established by adding PNP to FVIII-deficient plasma to final concentrations of 2.5% and 20% of FVIII levels, respectively^26^. PNP was used as the normal plasma model. All plasma models were depleted of MPs by centrifugation at 20,800 × *g* for 30 min or filtration through 0.1 µm Millipore filters (Merck Millipore, USA) before testing, as previously described^9^.

### Isolation of MPs from PNP

PNP was thawed at 37 °C and centrifuged at 20,800 × *g* for 30 min at 10 °C. The supernatant was discarded, and the remaining 50 µL were resuspended in 450 μL of phosphate-buffered saline (PBS), and centrifuged as above. The procedure was repeated twice to ensure removal of residual FVIII. The FVIII level of the isolated MPs was measured using a chromogenic two-stage FVIII activity assay and found to be undetectable.

### Flow cytometry analysis of MPs

The procedures were performed as previously described^27^. PS + MPs were identified using lactadherin-FITC. We defined PMPs as showing positive binding to both anti-CD42a-PE and anti-CD61-APC, regardless of binding to lactadherin. The enumeration of MPs was based on the acquisition rate of our instrument at a low flow rate (44 μL in 90 s)^28^. The amount of MPs used in coagulation assays (2, 3 and 7 × 10^4^ MPs/µL plasma) was based on the concentration of PMPs as the most abundant MP population in the plasma^29^.

### Calibrated automated thrombogram (CAT) assay

In the severe HA model, 20 μL of isolated MPs at three sequential concentrations (2, 3 and 7 × 10^4^ MPs/µL plasma) were added to 60 μL of FVIII-deficient plasma together with 20 μL of PPP-Reagent LOW or PBS. PPP-Reagent LOW contains TF and phospholipids with a final concentration of 1 pM and 4 μM, respectively. One concentration (2 × 10^4^ MPs/µL plasma) was selected to test the procoagulant effect of MPs in moderate and mild HA plasma models, as well as in PNP. The negative controls contained PBS instead of MPs or PPP-Reagent LOW. Thrombin generation was monitored at 20-second intervals for 90 min using a Fluoroscan Ascent fluorometer^30^. Data were analyzed using a thrombinoscope software package (version 3.0.0.29, Maastricht, the Netherlands). Endogenous thrombin potential (ETP, in nM thrombin × min), lag-time (in min) and peak thrombin generation (in nM thrombin) were determined.

### Overall hemostasis potential (OHP) assay

Fibrin formation and fibrinolysis were measured by using a modified OHP assay^31^. In order to test if MPs alone can trigger fibrin formation in the severe HA model, 85 μL of MPs at three selected concentrations (2, 3 and 7 × 10^4^ MPs/µL plasma) were added to 140 μL of FVIII-deficient plasma, and recalcified (final concentration, 13 mM) with or without tissue plasminogen activator (t-PA, 308 ng/mL). The negative control contained Tris buffer or MPs (7 × 10^4^ MPs/µL plasma) lysed with 0.25% TritonX-100 (incubated at RT for 15 min). In order to test the procoagulant effect of MPs in the presence of the OHP reagent (0.02 mM phospholipids and 0.04 U thrombin/mL), MP (2 × 10^4^ MPs/µL plasma) was added to plasma. The real-time absorbance change in the sample was monitored at λ = 405 nm, every 12 seconds for two hours. The area under the curve (absorbance vs. time) of fibrin formation (without t-PA) is defined as the overall coagulation potential, while the area under the curve of fibrinolysis (with t-PA) is the overall hemostasis potential (OHP) value.

### Blocking PS, TF or contact pathway activation

First, the PS present on MPs was blocked by incubation with lactadherin (100, 200 and 400 nM, Haematologic Technologies, USA) at RT for 30 min; the TF on MPs was blocked by incubation with anti-TF antibody (50 μg/mL, Sekisui Diagnostics, USA) at 37 °C for 15 min; contact pathway activation was inhibited by addition of corn trypsin inhibitor (CTI, 20 μg/mL, Haematologic Technologies, USA) to plasma. After these treatments, MPs were added to the mild HA plasma model, and thrombin generation and fibrin formation were measured using the CAT and OHP assays in the absence of phospholipids, TF or thrombin.

### PMP-associated procoagulant activity

Platelet concentrates were treated with or without thrombin receptor activation peptide-6 (TRAP6, Abcam, UK; 25 µM) at RT for 30 min. The remaining platelets were removed by centrifugation (1500 × *g*/5 min/15 °C, twice), and the supernatant was centrifuged to isolate PMPs. PMPs isolated from platelets with and without TRAP6 treatment were added to all plasma models, and tested by using the CAT and OHP assays.

### Scanning electron microscopy (SEM) analysis of fibrin gels

After the OHP assay was finished, fibrin clots were gently removed from plate-wells to micro-tubes by using an inoculating loop, washed with PBS (3 × 5 min), fixed in 2.5% glutaraldehyde and stored at 4 °C. Fibrin clot structure were observed using SEM as previously described^32^. Fiber thickness was measured manually using Fiji software^33^.

### Fibrin clot preparation for fluorescence imaging

Clots were formed on coverslips (#1.5, VWR international, USA) by adding a 20 µL mixture as in the OHP assay, with addition of Alexa Fluor 594-fibrinogen (0.5% of total fibrinogen, final, Thermo Fisher, USA). Clots were incubated with 5 µg/mL Abberior STAR 635 (Abberior Instrument, Germany)-conjugated anti-CD61 (VI-PL2, Thermo Fisher, USA), mounted with Mowiol^®^ 4–88 (Sigma-Aldrich, USA), sealed and stored at 4 °C in the dark until imaging.

### Confocal microscopy imaging and fibrin clot density analysis

Imaging was performed with a Leica SP8 laser scanning confocal microscope using a 63×/1.4 oil immersion HC PL APO lens. In each clot sample, optical sections were collected at eight randomly chosen areas (five in the periphery and three in the center). The size of each optical section was 100 × 100 µm (1248 × 1248 pixels) along the xy axes and 7.5 µm along the z axis from the first layer with fiber structure (starting from the coverslip), then divided into 0.3-µm sections. The images were analyzed using Fiji software^33^, and the fibrin clot density was calculated using the vessel density plug-in^34^ (https://imagej.net/Vessel_Analysis).

### Stimulated emission depletion microscopy (STED) imaging and analysis

STED micrographs were acquired using an Abberior Instruments setup (Abberior Instruments, Germany) with a Leica 100×, NA 1.4 objective. Depletion was performed at 775 nm for the two lasers at 594 nm and 637 nm, respectively (additional details see Supplementary data). Micrographs were analyzed using Fiji^33^ and Imspector^35^ (http://www.imspector.de) software.

### Statistical analysis

SPSS statistical software (version 25, IBM corporation, USA) was used. Data are shown by mean ± standard error of mean (SEM). To compare two fibrin density and fiber thickness, we used the Mann–Whitney *U* test or Student’s *t* test, respectively. Statistically significant differences were defined by *p* values ≤ 0.05.

## Supplementary information

Supplementary data.
