# Supplementary material for: Phosphatidylserine positive microparticles improve hemostasis in in-vitro hemophilia A plasma models
Source: Sci Rep. 2020 May 12;10:7871. doi: 10.1038/s41598-020-64686-x (PMC7217932; doi:10.1038/s41598-020-64686-x)
Supplement: Supplementary file 1 — Supplementary data. [file 41598_2020_64686_MOESM1_ESM.docx]

**Phosphatidylserine positive microparticles improve hemostasis in *in-vitro* hemophilia A plasma models**

Yanan Zong,^1*^ Iva Pruner,^1^ Aleksandra Antovic,^2.3^ Apostolos Taxiarchis,^1^ Zara Pons Vila,^1^ Nida Soutari,^4^ Fariborz Mobarrez,^5^ Roza Chaireti,^1,6^ Jerker Widengren,^7^ Joachim Piguet,^7^ and Jovan P. Antovic^1,4*^

^1^Department of Molecular Medicine & Surgery, Karolinska Institutet, Stockholm, Sweden; ^2^Department of Medicine, Division of Rheumatology, Karolinska Institutet, Stockholm, Sweden; ^3^Academic Specialist Center, Center for Rheumatology, Stockholm Health Services, Stockholm, Sweden; ^4^Coagulation, Clinical Chemistry, Karolinska University Hospital, Stockholm, Sweden; ^5^Department of Medical Sciences, Uppsala University, Akademiska Hospital, Uppsala, Sweden; ^6^Department of Hematology, Karolinska University Hospital, Stockholm, Sweden; and ^7^Experimental Biomolecular Physics, Department of Applied Physics, KTH Royal Institute of Technology, Stockholm, Sweden.

**Corresponding author:**

**Yanan Zong**

**Department of Molecular Medicine and Surgery, Karolinska Institutet
phone: +46 722717655
e-mail: yanan.zong@ki.se**

&

**Jovan P. Antovic MD, PhD, Associate professor
Department of Molecular Medicine and Surgery, Karolinska Institutet; Coagulation, Clinical Chemistry, Karolinska University Hospital, Stockholm, Sweden
Phone: +46 8 517 75637**

**Fax: +46 8 310 376
e-mail: jovan.antovic@ki.se**

**Methods**

**Stimulated emission depletion microscopy (STED) imaging and analysis**

STED micrographs were acquired using an Abberior Instruments setup, built on an Olympus IX83 stand, with an Abberior Quad scanner (Abberior Instruments, Germany), and modified for two-color STED imaging. Excitation was provided by two fiber-coupled diode lasers, pulsed at 20 MHz, one emitting at 637 nm (LDH-DC, PicoQuant AG, Germany), and the other at 594 nm (Abberior Instruments). Excitation alternated between the two lasers and the detection channels were gated to minimize crosstalk between channels. The beam of a pulsed fiber-coupled laser, PFL-P-30-775-B1R (MPB), emitting at 775 nm with a repetition rate of 40 MHz and a pulse width of 1.2 ns (1.2 W, 30 nJ pulse energy), provided simulated emission and was shaped by a phase plate (VPP-1c, RPC Photonics) into a donut profile. The three overlapping laser beams were focused by a Leica 100×, NA 1.4 objective into the sample. Fluorescence was collected through the same objective, focused onto a motorized pinhole (MPH16, Thorlabs), split by a dichroic mirror into two channels/paths, which then passed through an emission filter, FF01-615/20 and FF02-685/40-25 (Semrock) for the orange (fibrin) and red channels (microparticles) respectively. A common IR filter (FF01-775/SP-25; Semrock) was used to remove the scattered light from the STED laser. The signal was then detected by a single photon detector (SPCM-AQRH-13; Excelitas Technologies) for each channel. Image acquisition was controlled by Imspector (Abberior Instruments, Germany).

**Results**

**Characterization of MPs by flow cytometry**

The majority of events (approx. 90%) were detected within the gate P1, determined by Megamix-Plus SSC beads as previously described (Supplementary **Fig. 1a**; ^13^). Some events disappeared after treatment with 0.25% TritonX-100, confirming that these indeed corresponded to membrane vesicles (Supplementary **Fig. 1b).** PMPs (CD42a and CD61 double positive events) were almost completely removed by TritonX-100 (< 0.01% remained, Q2 in Supplementary **Fig. 1c-d**). The majority of PMPs contained PS on their surface, as detected by lactadherin-FITC binding (Supplementary **Fig. 1e,** Q2-1), but TF+ PMPs were negligible (Supplementary **Fig. 1f**, Q2-2, < 0.01%). Similarly, leukocyte-derived MPs (anti-CD45-APC) and endothelial cell-derived MPs (anti-CD144-PE) were negligible as well (data not shown).

**Figures**
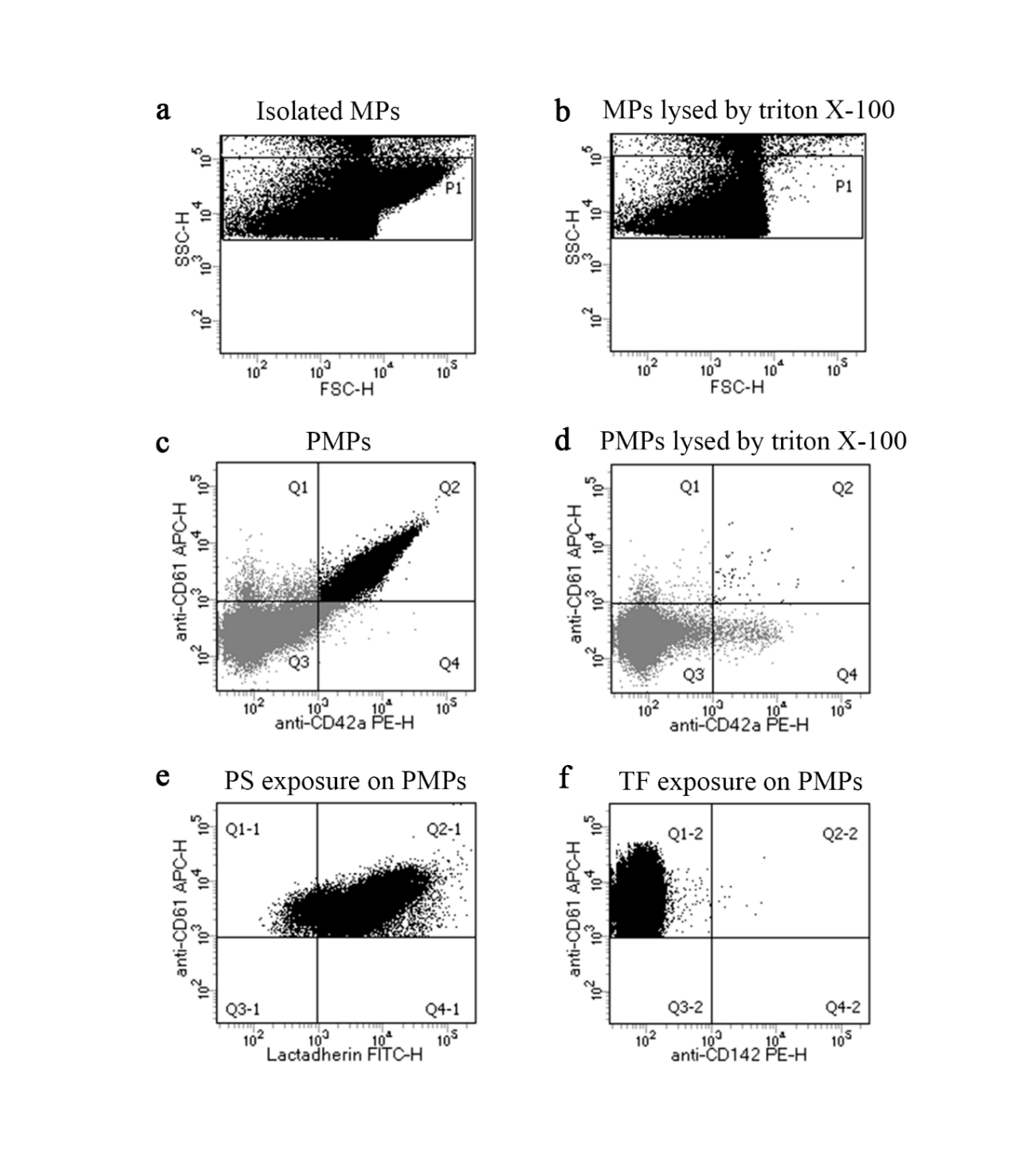


**Supplementary Figure 1. Characterization of MPs using flow cytometry**. a) MPs in the FSC vs. SSC dot plot: gate P1 was set by 0.16–0.5 µm standard Megamix SSC-Plus beads (corresponding to 0.3–1.0 µm Megamix FSC beads^19^); events in the gate represent the size range of MPs; b) In the FSC vs. SSC dot plot, events decreased after lysing MPs with 0.25% TritonX-100; c) MPs derived from platelets (PMPs) were identified as anti-CD42a-PE- and anti-CD61-APC-positive events (Q2, dark). The negative events are shown in Q3 (gray); d) PMPs were destroyed after treatment with TritonX-100; e, f) Exposure of PS (e) and TF (f) on PMPs, identified by binding of lactadherin and anti-CD142, respectively.

**
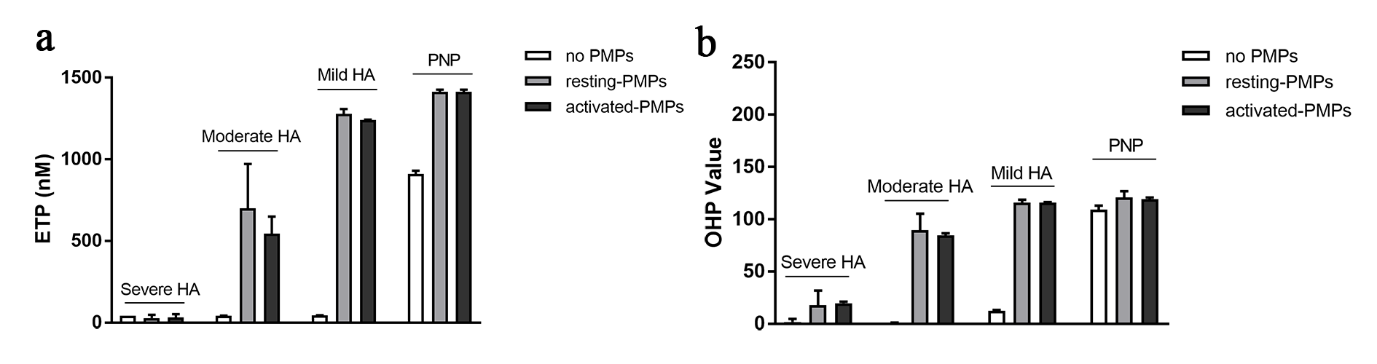
Supplementary Figure 2. The procoagulant effect of PMPs isolated from platelets with and without TRAP6 treatment in different plasma models**: a) endogenous thrombin potential (ETP) values determined by the CAT assay, and b) OHP values. Experiments performed in the absence of CAT and OHP reagent, n=triplicate wells/experiment.


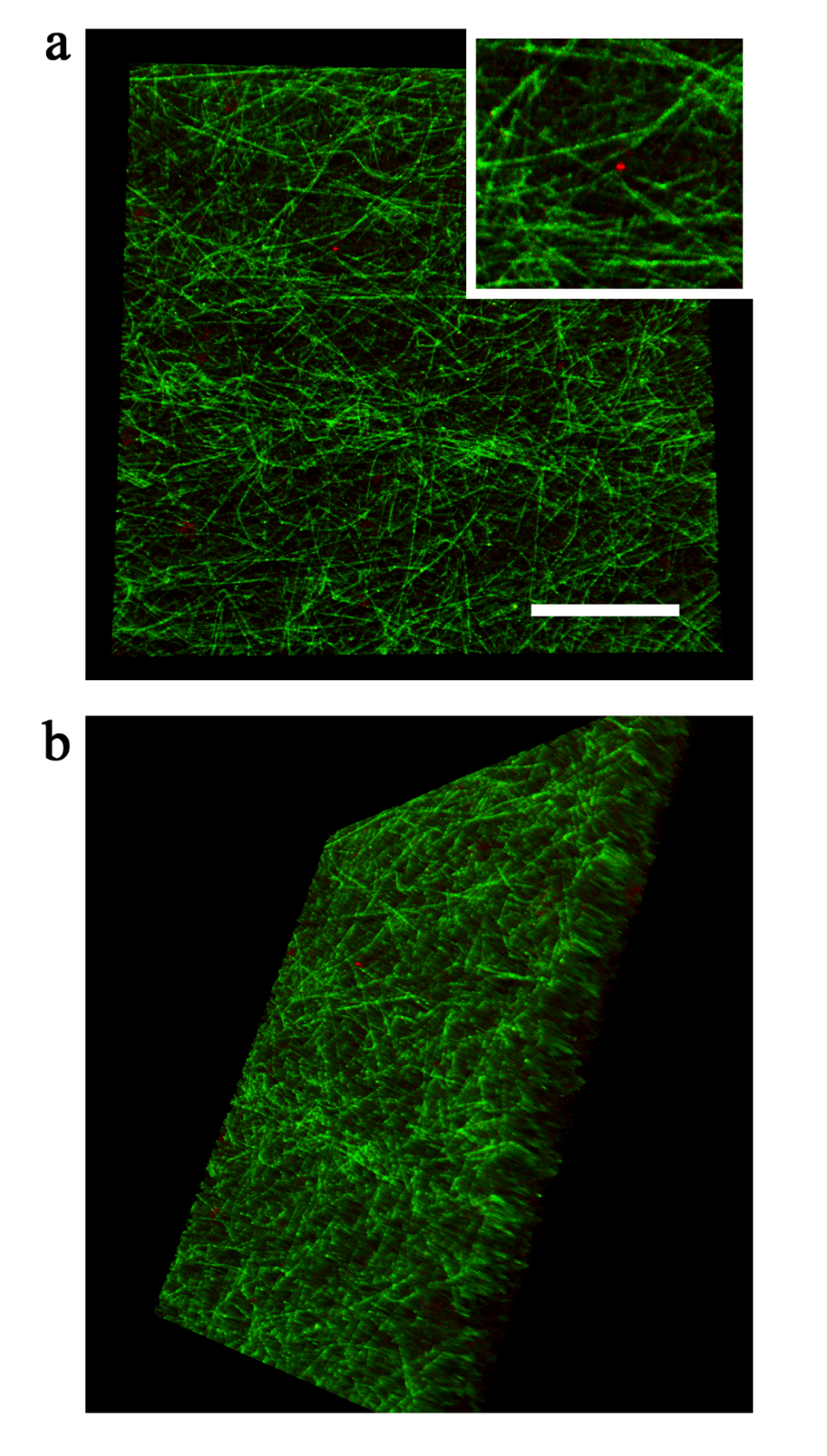


**Supplementary Figure 3. Incorporation of MPs in the three-dimensional fibrin clot.** a), top view, inset in (a) highlights one MP attached to fiber; b), rotated view, fibrin (green), PMP (red). The clot was form using severe HA plasma model with addition of MPs (2 × 10^4^ MPs/µL plasma), OHP reagent and calcium. The volume of the clot shown was 99.74 µm × 99.74 µm × 7.51 µm. Bar = 25 µm.


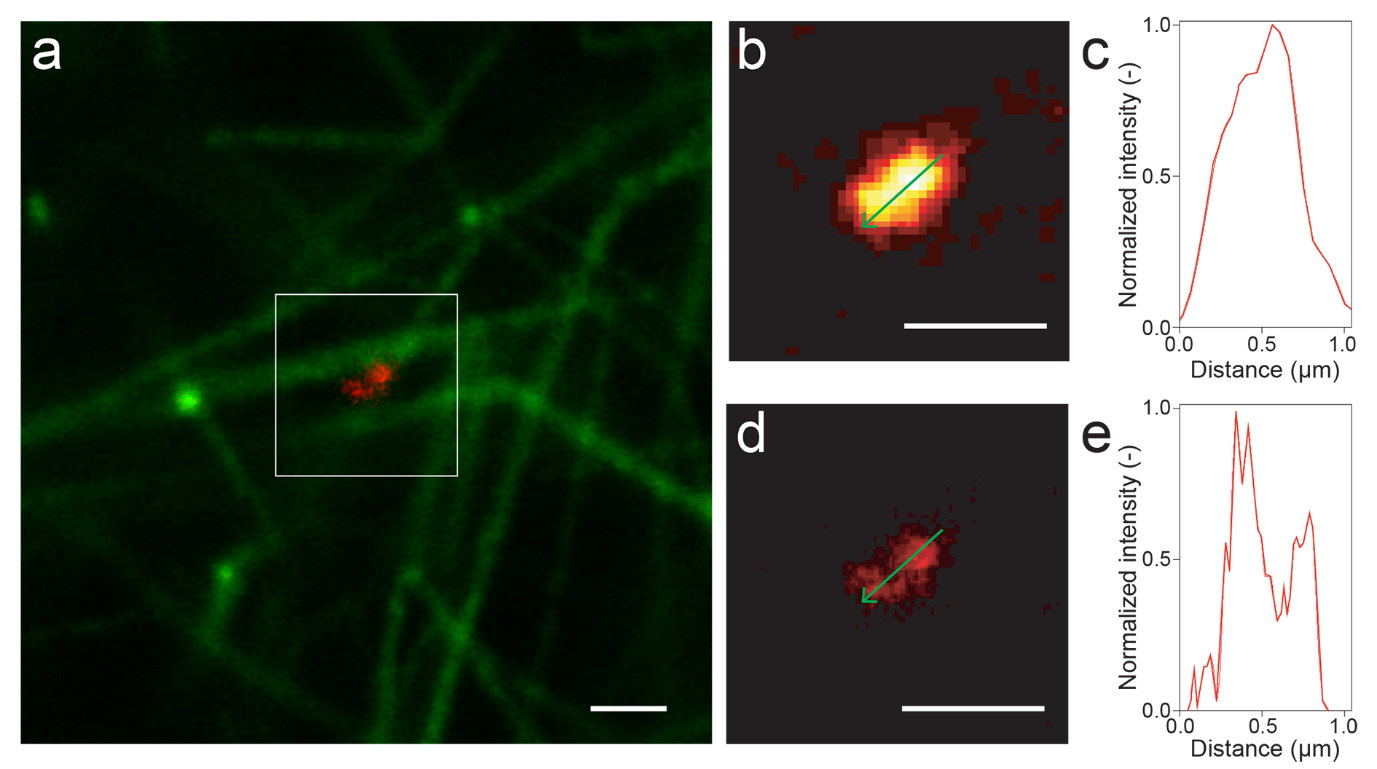


**Supplementary Figure 4. Two neighboring MPs identified by STED, incorrectly identified as a single MP by standard confocal microscopy.** a) STED image, MPs (red) attached to fibrin fibers (green); b, c) standard confocal microscopy image and intensity profile of the MP signal across the arrow drawn in (b); d, e) STED image and intensity profile. Scale bar = 1µm.
